# A Higher Proportion of Metabolic Syndrome in Chinese Subjects with Sleep-Disordered Breathing: A Case-Control Study Based on Electrocardiogram-Derived Sleep Analysis

**DOI:** 10.1371/journal.pone.0169394

**Published:** 2017-01-12

**Authors:** Ping-Huei Tseng, Pei-Lin Lee, Wei-Chung Hsu, Yan Ma, Yi-Chia Lee, Han-Mo Chiu, Yi-Lwun Ho, Ming-Fong Chen, Ming-Shiang Wu, Chung-Kang Peng

**Affiliations:** 1 Department of Internal Medicine, National Taiwan University Hospital, Taipei, Taiwan; 2 Center of Sleep Disorder, National Taiwan University Hospital, Taipei, Taiwan; 3 Department of Otolaryngology, National Taiwan University Hospital, Taipei, Taiwan; 4 Division of Interdisciplinary Medicine and Biotechnology, Beth Israel Deaconess Medical Center, Harvard Medical School, Boston, MA, United States of America; Charité - Universitätsmedizin Berlin, GERMANY

## Abstract

**Objective:**

The prevalence of metabolic syndrome (MS) has increased rapidly in Taiwan and worldwide. We aim to determine the association between sleep-disordered breathing (SDB) and MS in a Chinese general population.

**Methods:**

This case-control study recruited subjects who have undergone a prospective electrocardiogram-based cardiopulmonary coupling (CPC) sleep spectrogram as part of the periodic health check-ups at the National Taiwan University Hospital. Comprehensive anthropometrics, blood biochemistry, prevalence of MS and its individual components were compared with Bonferroni correction between 40 subjects with SDB, defined as the CPC-derived apnea–hypopnea index (CPC-AHI) >5 event/hour and 80 age- and sex-matched controls, defined as CPC-AHI <1 event/hour. MS was diagnosed based on the Adult Treatment Panel III, with a modification of waist circumference for Asians.

**Results:**

Subjects with SDB were more obese with larger waist circumferences (95.1±12.9 vs. 87.3±6.9, *P* < .001) and borderline higher BMI (27.0±4.9 vs. 24.3±2.5, *P* = .002). Waist circumference was independently associated with the presence of SDB after adjustment for BMI, systolic blood pressure and fasting blood glucose in multiple regression analyses. Subjects with SDB had a higher prevalence of central obesity (72.5% vs. 42.5%, *P* = .002), hyperglycemia (45.0% vs. 26.3%, *P* = .04), MS (45.0% vs. 22.5%, *P* = .01) and number of MS components (2.4 ± 1.6 vs. 1.7 ± 1.4, *P* = .01) than the control group. Waist circumference was significantly correlated with both CPC-AHI (r = .492, *P* = .0013) and PSG-AHI (r = .699, *P* < .0001) in the SDB group.

**Conclusions:**

SDB was associated with a higher prevalence of MS and its individual components, notably central obesity, in a Chinese general population. Large-scale screening of high risk population with MS to identify subjects with SDB for appropriate management is warranted.

## Introduction

Sleep-disordered breathing (SDB) and its most severe form, obstructive sleep apnea (OSA), are characterized by repeated pauses of breathing during sleep, leading to intermittent hypoxia and sleep fragmentation.[1] SDB/OSA has been associated with cardiovascular events, stroke, neurocognitive impairment, metabolic dysregulation and impairment of quality of life.[2–5] The incidence and prevalence of SDB has increased rapidly worldwide in recent years and imposes a substantial burden in respective health-care system.[6] Previous studies have identified several risk factors associated with SDB, including age, male gender, smoking and various components of metabolic syndrome (MS), especially central obesity and insulin resistance.[7–11]

Nowadays, the prevalence of the MS has been increasing rapidly in Taiwan and other Asian countries and appears to resemble that among the Western populations because of the westernization of diet and life style.[12] Although Lam and colleagues have found MS as an independent determinant of OSA in community-based Chinese adults in Hong Kong, studies regarding the association of SDB and MS remain limited in other Chinese populations.[13] Currently, polysomnography (PSG) is the gold standard for diagnosing OSA but it is costly, cumbersome, and practically unsuitable to large-scale population screening. Recently, with the great advances of nonlinear dynamics and statistical physics, there are several novel methods to study the simultaneously but independently existing forms of cardio-respiratory coupling solely based on electrocardiography (ECG) data, including cardio-respiratory synchronization, respiratory sinus arrhythmia (RSA) and cardio-respiratory time delay stability (TDS).[14–17] Among them, cardiopulmonary coupling (CPC) sleep spectrogram analysis derived from a continuous single-channel ECG recording, has been developed to quantify the objective features of sleep and further diagnose SDB.[18–20] In addition, previous studies have found such an ECG-based approach to be cost-efficient and provide clinically useful insight into abnormal sleep in various patient populations.[21, 22] Therefore, from January 2012, SDB screening with the ECG-based CPC analysis has been incorporated into the routine health check-up program for the general population in our institute. Subjects with identified SDB were further referred to our sleep center for detailed evaluation and management. With this prospective comprehensive evaluation of demographic and metabolic profile, as well as the subjective and objective of sleep evaluation, this pilot study aimed to investigate (1) the clinical characteristics of SDB subjects, and (2) the association between SDB and MS and its various components in the Chinese general population undergoing periodic health check-ups.

## Material and Methods

### Ethics statement

This prospective cross-sectional study was conducted in accordance with the principles of the Declaration of Helsinki, and was approved by the ethical committee of National Taiwan University Hospital (No. 201006037R). All participants gave their written informed consent prior to participating in the study.

### Study design and subject evaluation

From January 2012, all subjects aged equal to or greater than 20 years who underwent a self-paid health check-up at the Health Management Center of National Taiwan University Hospital were invited to participate in the SDB- screening program with an ECG-based CPC analysis. Attendees of the health check-up program in our institute were recruited through advertising messages for health-promotion purposes from the general population and therefore the participants did not belong to any particular socio-economic class or share a unifying form of employment. The standard protocol of our health check-up program consisted of a self-administered questionnaire, face-to-face interview by an internal medicine physician, physical examination, blood biochemical analysis, plain radiography for chest and abdomen, abdominal ultrasonography, upper endoscopy and colonoscopy. [23–25] Subjects who were found to have SDB by CPC analysis were referred to our sleep center for further evaluation, including a detailed history taking, local examination by an ear, nose and throat specialist, cranial x-ray and an overnight polysomnographic sleep study to confirm the presence of SDB and quantify its severity. Subjects with atrial fibrillation, use of ventricular pacing, severe comorbidities, such as congestive heart failure, symptomatic coronary heart disease, uncontrolled pulmonary disease, chronic renal failure, or pregnancy were excluded from the study.

### Systemic assessment of common risk factors of metabolic syndrome

All subjects underwent evaluation of cardiovascular and metabolic risk factors, including smoking, alcohol consumption, anthropometric measures, such as body mass index (BMI) and waist circumference, hypertension, hyperglycemia and dyslipidemia. Current drinkers were defined as those with at least one drink per week.[26] Waist circumference was measured at the level of the umbilicus at minimal respiration. Blood pressures measurement and blood tests were performed at 8:00–8:30 AM before taking any medication. All the blood pressure and anthropometric measurements were performed by trained and certified nurses. The laboratory tests, including fasting blood glucose, total cholesterol, high-density lipoprotein (HDL) cholesterol, low-density lipoprotein (LDL) cholesterol, triglyceride and uric acid levels, have both internal and external quality control procedures accredited by the Taiwan Society of Laboratory Medicine twice a year.

MS was diagnosed according to the criteria defined in the Adult Treatment Panel III, with a modification of waist circumference as appropriate for Asians.[27] Participants were diagnosed as having MS if they met three or more of the following five criteria: (1) waist circumference ≥90 cm in men and ≥80 cm in women; (2) systolic and/or diastolic blood pressure ≥ 130/85 mmHg or taking blood pressure-lowering medications; (3) fasting blood glucose concentration ≥100 mg/dL or taking hypoglycemic medications; (4) fasting triglyceride concentration ≥150 mg/dL; and (5) HDL concentration <40 mg/dL in men and <50 mg/dL in women.

### Assessment of sleep quality

Pittsburgh Sleep Quality Index (PSQI) was included in our standard questionnaires to assess sleep quality and quantity in all subjects.[28] This validated questionnaire consists of 19 items which generates seven components to assess sleep quality and disturbances over the previous one month, including subjective sleep quality, sleep latency, sleep duration, habitual sleep efficiency, sleep disturbance, use of sleep medication and daytime dysfunction. The score of each component ranges from 0 to 3. The sum of these seven component scores provides a global PSQI score which ranges from 0 to 21. A higher global PSQI score indicates poorer sleep quality.

### Continuous ambulatory ECG recordings

All subjects were monitored with an ambulatory single-channel ECG monitor (DynaDx Corporation, Taipei, Taiwan) at home one week after completing their routine health check-ups. Subjects were instructed to keep their usual time to bed and getting up where it was documented on their sleep diary. ECG recording started from their bed time and ended upon awakening in the next morning. The device was then removed and returned to the institute. The recordings were processed by standard automated algorithm to generate sleep spectrogram.

### Cardiopulmonary coupling analysis and sleep spectrograms

The CPC technique is based on a continuous ECG signal and uses the Fourier Transform to analyze 2 features of the signals: 1) heart rate variability and 2) the fluctuations in R-wave amplitude induced by respiration. These signals tend to have two basic patterns: a high-frequency component due to physiological sinus arrhythmia that reflects breath-to-breath fluctuations and a low-frequency component that reflected cyclic variation across multiple breaths. Quantification of cardiac and respiratory interactions involves calculating the cross-power and coherence between these 2 signals. Three physiological sleep states are obtained from the ECG-based CPC analysis, including restful (indicated by high frequency coupling), disturbed (indicated by low frequency coupling), and REM/wakeful states (indicated by very low frequency coupling).[18, 29, 30] With the additional information about sleep and wakefulness times reported by the subject, main sleep-related parameters in the present study included: bed to the occurrence of restful sleep, defined as the time of falling in bed to the time that restful sleep occurs; total sleep time, defined as the time of falling in bed to the time getting out of the bed; restful sleep ratio, defined as the ratio of restful sleep time to total sleep time; disturbed sleep ratio, defined as the ratio of disturbed sleep time to total sleep time; wake/rapid eye movement (REM) ratio, defined as the ratio of wake/REM time to total sleep time; and apnea/hypopnea index (CPC-AHI), defined by duration and mean frequency of the low frequency coupling periods, and expressed as number/h.

### Statistical analysis

In the prior study by Thomas et al., there was a good correlation of the ECG-based algorithm for detecting the sleep apnea/hyponea with the expert human scoring of the PhysioNet Sleep Apnea Database (overall correlation coefficient r = .88, *P* < .01), and subjects with no apnea (apnea time = 0 during the recording) were found to have a AHI of .04±.10/h by full laboratory polysomnograms.[29] Therefore, in this case-control study, we recruited subjects who were suspected to have SDB through the ECG-based CPC analysis (defined as CPC-AHI>5) and were confirmed by overnight PSG in our sleep center as the case group.[31] Twice of age- and sex-matched controls without definite evidence of SDB (more strictly defined as CPC-AHI<1) were randomly selected to increase the validity of the present study (SDB case: control = 1:2).[29]

First, we compared basic demographic data, anthropometric measurements, metabolic profile and MS components between subjects with and without SDB. Second, we assessed the pairwise relationships between SDB and MS and its various components with *Pearson* correlation. Logistic regression analyses were used to determine whether MS and/or its various components were significant factors associated with the presence of SDB. Continuous data were expressed as the mean ± standard deviation (SD) and compared by Student *t* test or non-parametric test, when appropriate. Categorical data were expressed as percentage and analysed by *Pearson* χ^2^ tests or Fisher exact tests, as appropriate. For multiple comparisons on the various metabolic and sleep parameters, Bonferroni correction was applied to decrease the false discovery rate. All statistical analyses were performed using SPSS 16 (SPSS, Inc., Chicago, IL, USA).

## Results

### Participant characteristics

Finally, 40 SDB subjects and 80 controls were recruited for analysis. As illustrated in Table 1, the mean age of SDB subjects was 52.5 years (range, 36–73) and male gender was predominant (82.5%). Compared to the control group, SDB subjects were more obese with larger waist circumference (95.1±12.9 vs. 87.3±6.9, *P* < .001) and borderline higher BMI (27.0±4.9 vs. 24.3±2.5, *P* = .002). Social habits, such as smoking, alcohol consumption and habitual exercise, as well as previous medical history, were not significantly different between these two groups. Abnormal metabolic profile, including elevated systolic blood pressure and fasting hyperglycemia, appeared to be positively associated with the presence of SDB, albeit not statistically significant after Bonferroni correction.

**Table 1:** Comparison of demographic characteristics, medical histories, anthropometrics, laboratory findings, sleep problems and Pittsburgh Sleep Quality Index between subjects with sleep-disordered breathing and the control group.

Characteristics	SDB	Control	*P*-value*
Case number	40	80	
Age, yr	52.5 ± 8.3	53.1 ± 8.8	.72
Male gender	33 (82.5)	66 (82.5)	>.99
**Social habits**			
Current drinker	4 (10.0)	15 (18.8)	.29
Current smoker	4 (10.0)	13 (16.3)	.41
Habitual exercise	12 (30)	32 (40)	.58
**Medical history**			
Hypertension	13 (32.5)	15 (18.8)	.09
Diabetes	9 (22.5)	8 (10.0)	.06
Hyperlipidemia	3 (7.5)	14 (17.5)	.13
Hyperuricemia	4 (10.0)	6 (7.5)	.64
Coronary artery disease	1 (2.5)	3 (3.8)	>.99
Anti-hypertensive agent use	11 (27.5)	16 (20.0)	.35
Hypoglycemic agent use	8 (20.0)	6 (7.5)	.04
Anxiolytics/hypnotics use	2 (5.0)	8 (10.0)	.35
**Anthropometrics**			
BMI, kg/m^2^	27.0 ± 4.9	24.3 ± 2.5	.002
Waist circumference, cm	95.1 ± 12.9	87.3 ± 6.9	.001
Male	96.4 ± 11.4	87.9 ± 6.7	< .001
Female	89.1 ± 18.2	84.6 ± 7.4	.42
**Metabolic profile**			
SBP, mm Hg	128.8 ± 18.0	122.1 ± 14.0	.02
DBP, mm Hg	76.7 ± 10.9	73.3 ± 10.0	.08
Fasting blood glucose, mg/dL	107.2 ± 24.6	97.0 ± 15.3	.02
HbA1c, %	5.9 ± 0.6	5.7 ± 0.6	.11
Triglycerides, mg/dL	137.6 ± 58.0	134.4 ± 82.2	.82
Total cholesterol, mg/dL	194.9 ± 25.5	189.4 ± 34.0	.36
HDL, mg/dL	45.1 ± 13.3	47.3 ± 9.5	.31
LDL, mg/dL	126.6 ± 20.1	119.6 ± 31.4	.14
Uric acid, mg/dL	6.6 ± 1.5	6.1 ± 1.3	.11
hs-CRP, mg/L	0.28 ± 0.43	0.14 ± 0.15	.05
**PSQI**			
Sleep duration	0.90 ± 0.72	1.16 ± 0.85	.09
Time to fall asleep	0.55 ± 0.68	1.02 ± 0.76	.001
Sleep efficiency	0.48 ± 0.82	1.04 ± 1.01	.003
Trouble sleeping	2.03 ± 1.05	1.85 ± 1.08	.39
Sleep quality	1.35 ± 0.83	1.61 ± 0.72	.07
Medicine use to help sleep	0.23 ± 0.66	0.48 ± 0.97	.09
Trouble in staying awake	0.70 ± 0.91	0.68 ± 0.87	.88
Total PSQI scores	6.20 ± 3.77	7.84 ± 3.94	.03

Data are presented as mean ± standard deviation or number (percentage).

Abbreviation: SDB, sleep-disordered breathing; BMI, body mass index; SBP, systolic blood pressure; DBP, diastolic blood pressure; HDL, high-density lipoprotein; LDL, low-density lipoprotein; hs-CRP, high-sensitivity C-reactive protein; PSQI, Pittsburgh Sleep Quality Index.

Comparisons between quantitative data using Student *t*-tests and categorical data using Pearson χ^2^ tests or Fisher exact tests when appropriate.

*P < .0016 (0.05/31 = 0.0016) is considered statistically significant to correct for multiple comparisons.

### Sleep-related parameters between SDB and control group

**Fig 1:**
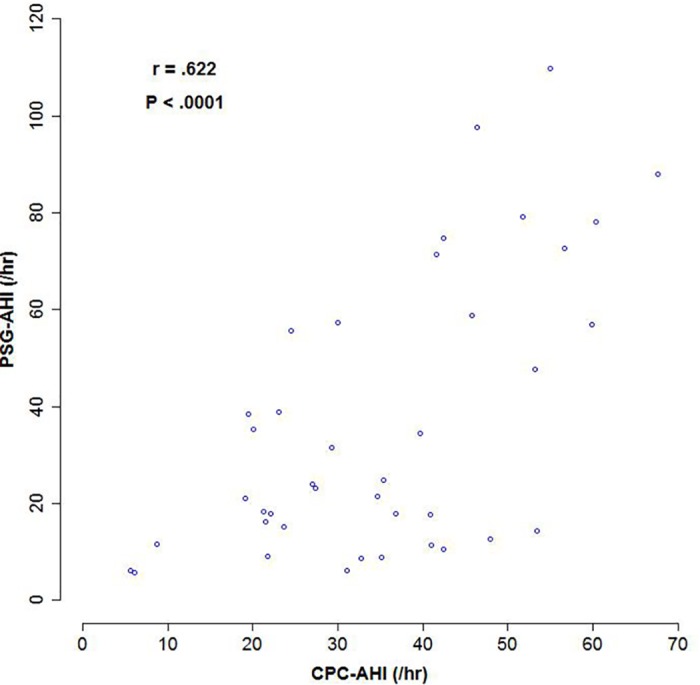
Correlation between CPC-AHI and PSG-AHI. Pearson correlation between the apnea-hypopnea index (AHI) obtained by cardiopulmonary coupling analysis (CPC-AHI) and by polysomnography (PSG-AHI) in 40 patients with identified sleep-disordered breathing.

Based on the self-reported sleep questionnaire PSQI, SDB patients had lower scores on the dimension of “time to fall asleep” and “sleep efficiency”, as well as the total PSQI scores, suggesting a better subjective sleep quality than the control group. We further compared various sleep-related parameters obtained from ECG-based CPC analysis between the SDB group and the control group. As shown in Table 2, SDB subjects had significant longer bed-to-restful-sleep time, lower restful sleep ratio and wake/REM ratio, as well as a higher disturbed sleep ratio and apnea ratio. For the SDB group who had both the CPC study and polysomnography, the AHI obtained by CPC analysis (CPC-AHI) was highly correlated with the PSG-AHI (r = .622, *P* < .001), as shown in Fig 1.

**Table 2:** Comparison of sleep-related parameters obtained from electrocardiogram-based cardiopulmonary coupling analysis between subjects with sleep-disordered breathing and the control group.

Sleep parameter from CPC	SDB (n = 40)	Control (n = 80)	*P*-value*
Bed to restful sleep time, min	60.1 ± 81.9	18.7 ± 24.4	.003
Total sleep time, hr	7.5 ± 1.4	7.3 ± 1.1	.76
Restful sleep ratio, %	18.4 ± 14.6	44.9 ± 18.5	< .001
Disturbed sleep ratio, %	65.5 ± 14.4	30.1 ± 16.2	< .001
Wake/REM ratio, %	14.8 ± 7.5	23.6 ± 10.3	< .001
CPC-AHI, /hr	33.4 ± 16.1	0.006 ± 0.06	< .001

Data are presented as mean ± standard deviation or number (percentage).

Abbreviation: SDB, sleep-disordered breathing; REM, rapid eye movement; CPC, cardiopulmonary coupling; AHI, apnea-hypopnea index.

Comparisons between quantitative data using Student *t*-tests and categorical data using Pearson χ^2^ tests or Fisher exact tests when appropriate.

*P < .008 (0.05/6 = 0.008) is considered statistically significant to correct for multiple comparisons.

### Logistic regression analysis

As shown in Table 3, univariate analyses confirmed several traditional risk factors associated with SDB, including BMI, waist circumference, systolic pressure and fasting blood glucose. Using multivariate analyses, only waist circumference remained independently associated with the presence of SDB (adjusted odds ratio [OR] = 1.10; 95% confidence interval [CI] = 1.04–1.16; *P* = .001).

**Table 3:** Logistic regression analysis for sleep-disordered breathing based on metabolic parameters.

	Univariate analyses			Multivariate analyses		
Variables	Crude OR	95% CI	*P*-value*	Adjusted OR	95% CI	*P*-value*
BMI	1.27	1.10–1.46	.001			
Waist circumference	1.10	1.04–1.16	< .001	1.10	1.04–1.16	.001
Systolic blood pressure	1.03	1.00–1.05	.03			
Fasting blood glucose	1.03	1.01–1.05	.01			

Abbreviation: OR, odds ratio; CI, confidence interval; BMI, body mass index.

Adjusted for BMI, waist circumference, systolic pressure, and fasting blood glucose.

*P < .05 indicates statistical significance.

### Association between SDB and MS

**Fig 2:**
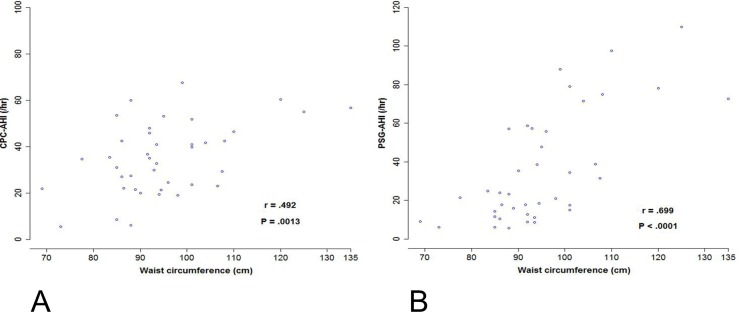
Correlation between the waist circumference and apnea-hypopnea index. Pearson correlation between the waist circumference and apnea-hypopnea index (AHI) obtained by (A) cardiopulmonary coupling analysis (CPC-AHI) and by (B) polysomnography (PSG-AHI), respectively, in 40 subjects with sleep-disordered breathing.

We further compared the prevalence rates of MS and individual MS components between SDB subjects and the controls. As shown in Table 4, SDB subjects had a significantly higher prevalence of MS (45.0% vs. 22.5%, OR = 2.8, 95% CI = 1.2–6.3, *P* = .01) and number of MS components (2.4±1.6 vs. 1.7±1.4, *P* = .009). Among the individual MS components, subjects with SDB were associated with higher prevalence of central obesity (72.5% vs. 42.5%, OR = 3.6, 95% CI = 1.6–8.1, *P* = .002) and fasting hyperglycemia (45.0% vs. 26.3%, OR = 2.3, 95% CI = 1.0–5.1, *P* = .002). Since central obesity was independently associated with the presence of SDB, we further explored the relationship between the degree of central obesity (waist circumference) and SDB (AHI). As shown in Fig 2, waist circumference was significantly correlated with both CPC-AHI (r = .492, *P* = .0013) and PSG-AHI (r = .699, *P* < .0001) in the SDB group.

**Table 4:** Comparison of metabolic syndrome and its various components between subjects with sleep-disordered breathing and the control group.

Metabolic syndrome component	SDB (n = 40)	Control (n = 80)	OR (CI)	*P*-value*
Waist circumference (central obesity)	29 (72.5)	34 (42.5)	3.6 (1.6–8.1)	.002
High blood pressure	19 (47.5)	29 (36.3)	0.6 (0.7–3.4)	.23
Low HDL	14 (35.0)	21 (26.3)	1.5 (0.7–3.4)	.32
Hyperglycemia	18 (45.0)	21 (26.3)	2.3 (1.0–5.1)	.04
Hypertriglyceridemia	17 (42.5)	29 (36.3)	1.3 (0.6–2.8)	.50
Number of MS components	2.4 ± 1.6	1.7 ± 1.4	-	.01
Metabolic syndrome	18 (45.0)	18 (22.5)	2.8 (1.2–6.3)	.01

Data are presented as mean ± standard deviation or number (percentage).

Abbreviation: OR, odds ratio; CI, confidence interval; HDL, high-density lipoprotein.

Comparisons between quantitative data using Student *t*-tests and categorical data using Pearson χ^2^ tests or Fisher exact tests when appropriate.

*P < .05 indicates statistical significance.

## Discussion

The key findings in the present study includes: (1) SDB was significantly associated with MS components, including obesity, hypertension, and fasting hyperglycemia in the Chinese population. (2) After adjusting for other common metabolic factors or MS components in multivariate logistic regression, waist circumference (central obesity) remained the independent determinant of SDB.

Epidemiological studies have reported a close relationship between SDB and MS.[32–35] Among the individual components of MS, central obesity has been considered to be a pivotal risk factor for both SDB and MS.[36, 37] While obesity contributes to the airway narrowing through fat deposition in the pharyngeal soft tissue,[38] central (abdominal) obesity may reduce the lung volume, decrease longitudinal tracheal traction forces and pharyngeal wall tension and therefore predispose to airway narrowing or collapse.[39] In the present study, we confirmed that subjects with SDB had higher BMI and waist circumference than the control group. Moreover, waist circumference, an index of central obesity, was independently associated with the presence of SDB after adjustment for BMI, systolic blood pressure and fasting blood glucose in multiple regression analyses. Our study also showed that subjects with SDB had a higher prevalence of MS and number of MS components than the control group. Previous studies focusing on the association of SDB or OSA and MS in the Chinese population remain scarce. In a prospective cross-sectional study from 255 community-dwelling volunteers in Hong Kong, the authors have identified several independent determinants of OSA, including age, gender, BMI and MS.[13] Similarly, in a recent study from Taiwan, Wu et al. also showed a high prevalence of MS (73.3%) in male bus drivers with PSG-proved OSA and found BMI to be the major contributing factor of OSA.[40] In the present study, we have matched the age and gender in the control group and we found that waist circumference was more closely associated with the presence of SDB than BMI was, an index of general obesity. Moreover, Lin et al. also found that OSA was independently associated with some components of MS, including dyslipidemia, hypertension and at least two of metabolic abnormalities in nonobese Chinese subjects from China.[41] These limited but important studies have highlighted the close association between MS and SDB/OSA in the Chinese population. Further studies to clarify the relative impact of these metabolic components, particularly central and general obesity, are warranted.

The independent association of SDB with insulin resistance, hyperglycemia and type 2 diabetes has also been confirmed in several epidemiological and clinic-based studies.[10, 42–44] However, obesity has also been found to be the major determinant in the development of insulin resistance and metabolic abnormalities in subjects with SDB.[11] In the present study, subjects in the SDB group have significant higher fasting blood glucose, higher prevalence of hyperglycemia and marginally higher prevalences of previously diagnosed diabetes and hypoglycemic agent use than the control group. However, such association became non-significant after adjusting for other MS factors such as BMI and waist circumference. Since the obesity and adiposity were less prominent in the Chinese and Asian populations than the Western populations, further large-scale longitudinal and interventional studies may be needed to elucidate the exact relationship between SDB and insulin resistance.

In the present study, SDB patients had lower scores on the dimension of “time to fall asleep” and “sleep efficiency”, as well as the global PSQI scores. The reasons why SDB subjects reported better sleep quality remain unclear. In a recent study, Scarlata et al. studied 254 subjects referred to a PSG evaluation and all patients were administered the PSQI before the procedure. The authors found the PSQI score had poor correlation with AHI in the pre-PSG assessment of people with suspected OSA. Hung et al. also found that subjects with MS have higher global PSQI scores and a higher risk of being poor sleepers in 3435 Chinese subjects who received a health examination.[45] However, objective measurement of sleep quality and quantity, such as PSG, was not used in that study and thus SDB cases could not be well identified. Future prospective studies incorporating both subjective sleep quality evaluation and objective sleep measurement may help to clarify the relationship between sleep quality and SDB/OSA in the Chinese population.

Strengths of the present study include a better selection of the appropriate age- and sex-matched control group without SDB through this ECG-based CPC analysis. Furthermore, all SDB cases detected by the ECG-based CPC analysis have been confirmed by a thorough evaluation, including an overnight PSG, in the sleep laboratory. Nevertheless, our study has several limitations. First, as the study was mainly carried out in the general population who received routine health check-up, the case number of SDB, especially the female cases, was relatively small, compared with studies carried out in the sleep center. In addition, multiple comparisons have been made between the control group and the SDB group on the various metabolic and sleep parameters and the false discovery could not be totally excluded based on the original significance level, that is, *P* < .05. We have thus applied the Bonferroni correction in our comparisons for the standard significance levels. Although several metabolic measures, such as elevated systolic blood pressure and fasting hyperglycemia, have become less statistically significant after such rigorous correction, future studies with larger sample size are needed to evaluate their exact impact on the SDB. Second, as our program was self-referred and self-funded, we cannot exclude the possibility that our participants might not readily represent a community population. Third, although we have performed rigorous analyses to evaluate the association of MS and its components with the presence of SDB, our cross-sectional design could not clarify the causal-effect relationship between MS and SDB and vice versa. Future interventional studies with exercise or weight loss to improve obesity or with medical/surgical treatment to downgrade the severity of SDB may help to elucidate the temporal relationship between SDB and MS/obesity. Finally, some factors including inflammatory and anti-inflammatory cytokines, adipocytokines, fasting insulin, homeostatic model assessment–insulin resistance and sympathetic activities were not measured in this study. The possible confounding effects by these factors cannot be totally excluded.

In conclusion, the present study demonstrated that SDB was significantly associated with MS in the Chinese population. Among the various MS components, central obesity, in terms of waist circumference, was the most crucial and independent determinant of SDB. With the ever-increasing prevalence of obesity and MS in the Chinese population, efforts toward screening of high risk subjects, early detection of subjects with SDB, and referral for confirmatory tests and appropriate medical or surgical management to improve their health outcomes and prevent the cardiovascular complications are warranted.

## Abbreviations

SDB: sleep-disordered breathing
MS: metabolic syndrome
CPC: cardiopulmonary coupling
AHI: apnea–hypopnea index
BMI: body mass index
OSA: obstructive sleep apnea
PSG: polysomnography
ECG: electrocardiography
HDL: high-density lipoprotein
LDL: low-density lipoprotein
PSQI: Pittsburgh Sleep Quality Index
REM: rapid eye movement
SD: standard deviation
OR: odds ratio
CI: confidence interval
